# DNA hypomethylation of the *OLFM1* gene in patients with depression

**DOI:** 10.3389/fneur.2026.1919737

**Published:** 2026-08-27

**Authors:** Chen Zhou, Lili Qing, Tiantian Zou, Ming Zhao, Xinrui Guo, Wensa Yin, Jia Wang, Yinlei Lei, Yinghan Li, Liping Hu, Linlin Liu, Shengjie Nie

**Affiliations:** 1 NHC Key Laboratory of Drug Addiction Medicine, School of Forensic Medicine, Kunming Medical University, Kunming, Yunnan, China; 2 Department of Anesthesiology, The Second Affiliated Hospital of Kunming Medical University, Kunming, Yunnan, China; 3 Mental Health Center of Yunnan Province, Kunming, Yunnan, China; 4 Kunming Yan’an Hospital, Kunming, Yunnan, China

**Keywords:** CpG sites, depression, DNA methylation, hypomethylation, *OLFM1*

## Abstract

**Objective:**

Depression is a heterogeneous psychiatric disorder and a growing public health concern, characterized by its high prevalence, recurrence rate, and association with suicide. There is evidence suggesting that both genetic susceptibility and environmental factors can regulate gene expression through DNA methylation, thereby influencing the occurrence and development of depression. The olfactory sensory neuropeptide 1 (*OLFM1*) protein is a risk factor for mental disorders. However, there are no reports yet regarding the correlation between the *OLFM1* gene and depression, nor have there been any studies on the association between *OLFM1* gene DNA methylation and depression.

**Methods:**

Genomic DNA was extracted from peripheral blood samples of patients with depression (*n* = 100) and healthy controls (*n* = 100) using the QIAamp DNA Blood Mini Kit. Subsequently, the extracted genomic DNA was subjected to bisulfite treatment using the EZ DNA Methylation-Gold™ kit. DNA methylation levels of 107 CpG sites in six fragments of *OLFM1* exon 1 and its downstream were detected by the Illumina HiSeq platform using MethylTarget™ technology.

**Results:**

Methylation levels across the overall *OLFM1* CpG island and its six fragments (*OLFM1*-1 to *OLFM1*-6) were significantly reduced in the depression group relative to controls. Analysis of the *OLFM1* gene fragments revealed that 84 of 107 CpG sites were significantly hypomethylated in depressed individuals. When patients were divided by sex, male patients displayed hypomethylation at 65 CpG sites, substantially more than the 37 sites found in females.

**Conclusion:**

*OLFM1* hypomethylation is associated with depression and may serve as a potential epigenetic biomarker.

## Introduction

Depression, a highly prevalent and debilitating psychiatric disorder, represents a significant global public health challenge, exerting profound impacts on individuals, families, and societal systems. Clinically, depression is characterized by persistent low mood, anhedonia (e.g., diminished ability to experience pleasure), cognitive dysfunction, and disturbances in sleep and appetite. Current epidemiological data from the World Health Organization indicated that depression affects approximately 350 million people worldwide (1), ranking among the top contributors to global disability-adjusted life years. In severe cases, these symptoms may escalate to suicidal ideation or behaviors (2), ultimately leading to significant functional impairment and increased mortality. Moreover, even with adequate dosage and duration of antidepressant treatment, 50 to 60% of patients still fail to achieve complete remission in clinical settings (3). Therefore, elucidating the pathogenic factors of depression and identifying novel, effective biomarkers are of strategic importance for its effective prevention and control.

Depression is currently explained by several hypotheses, including monoamine neurotransmitter deficiency (4), neurotrophic factor depletion (5), oxidative stress (6), and immune-inflammatory dysregulation (7). However, the effects of corresponding clinical treatment strategies based on these hypotheses are unsatisfactory. Increasing evidence has proved that neuronal development and synaptic function abnormalities constitute core pathological features of depression, participating in disease initiation and progression through multi-level regulatory dysfunctions (8, 9). Interestingly, olfactomedin 1 (*OLFM1*) plays important roles in neurodevelopment, synaptic plasticity, and neuronal survival (10, 11). Functionally, *OLFM1* encodes a secreted glycoprotein that is predominantly expressed in the central nervous system. Emerging evidence suggests that *OLFM1* was involved in various biological processes, such as cortical cell migration, neural crest formation, and neuronal differentiation (12, 13). A recent study confirmed that the decrease in serum *OLFM1* level was positively correlated with cognitive impairment (10). Nakaya et al. (14) found that knockdown of *OLFM1* caused abnormal behavior and brain dystrophy in mice. Another study confirmed that elevated *OLFM1* was observed in patients with Gilles de la Tourette syndrome (15), which was a neuropsychiatric disorder with strong genetic etiology. These findings indicated that *OLFM1* may play a significant role in psychiatric disorders, but its regulatory function in depression has not yet been investigated.

Currently, epigenetic alterations have been reported in individuals with depression and may serve as effective biomarkers used for the diagnosis and treatment of patients with depression (16). Of note, epigenetic modifications of gene expression provide a mechanistic framework for understanding the link between persistent effects of adverse life events and altered expression of psychiatric disorder-related genes (17, 18). Previous studies have confirmed that exposure to stress or traumatic life events (particularly during early life) was one of the risk factors for various mental illnesses such as depression (19, 20). Meanwhile, adverse childhood experiences are an important risk factor for developing depression (21). In recent years, DNA methylation has been one of the main forms of epigenetic modification and the most studied epigenetic mechanisms in depression. For example, abnormal DNA methylation (e.g., *NR3C1* and *BDNF*) was a risk factor for patients with depression (22, 23). Schiele et al. (24) reported that the altered DNA methylation patterns of *TIMP2*, *VDAC1*, or *SORL1* genes were associated with antidepressant treatment response in depression. Another study showed that elevated *BICD2* DNA methylation improved the depression-like behavior in a depression model mouse (25). Therefore, identifying genes with altered methylation patterns in depression is essential for understanding the molecular basis of the disorder and for developing novel therapeutic strategies. To date, the DNA methylation status of *OLFM1* gene in depression has not been reported.

To our knowledge, CpG island methylation in regions further upstream of the start codon influences gene expression and plays a role in depression progression. However, the association between the methylation level of the CpG island of the *OLFM1* gene and depression has not yet been studied. Since methylation of exon 1 and its downstream region can be regulated by both transcriptional and post-transcriptional mechanisms, we examined *OLFM1* methylation at this region via sequencing genomic DNA from depressed patients and healthy controls. We further analyzed the association between *OLFM1* methylation levels and sex, which helps elucidate the role of *OLFM1* epigenetic regulation in depression.

## Materials and methods

### Samples

A total of 200 participants were recruited for this study, including 100 patients with depression (33 males and 67 females) and 100 healthy controls (64 males and 36 females). Patients with depression were recruited from Yunnan Psychiatric Hospital. All patients were diagnosed by two independent psychiatrists according to the Diagnostic and Statistical Manual of Mental Disorders. The inclusion criteria for the depression group were patients who met the Diagnostic and Statistical Manual of Mental Disorders Fifth Edition (DSM-V) and the 17-item Hamilton rating scale for depression criteria. The exclusion criteria for patients with depression included: comorbid psychiatric disorders (e.g., bipolar disorder, schizophrenia), a history of neurological diseases (e.g., stroke, epilepsy), substance abuse within the past 6 months, and current use of psychotropic medications that may affect epigenetic profiles (e.g., valproate, lithium). Healthy controls were recruited from the CDC in Yunnan Province, with no personal or family history of psychiatric disorders, as confirmed by the Mini International Neuropsychiatric Interview. The demographic characteristics of all participants, who resided in Yunnan Province, China, are summarized in Table 1.

**Table 1:** Clinical pathologic characteristics.

Parameter	Control group (*n* = 100)	Depression group (*n* = 100)
Age (year)	34.74 ± 9.58	23.00 ± 14.92
BMI (kg/m^2^)	22.00 ± 2.70	22.70 ± 3.96
Educational level		
Low education	17	50
High education	83	50
Marital status		
Single	47	70
Married	53	30

Peripheral blood samples (3 mL) were collected from each participant in EDTA-containing tubes, stored at −80 °C within 2 h of collection, and processed for DNA extraction. This study was approved by the Ethics Committee of the Kunming Medical University (NO: KMMU2020MEC011), and written informed consent was obtained from all participants.

### DNA extraction and bisulfite transformation

Genomic DNA was extracted from peripheral blood samples using the QIAamp DNA Blood Mini Kit (Qiagen, Germany) following the manufacturer’s protocol. The quantity and purity of extracted DNA were assessed using a NanoDrop 2000 spectrophotometer (Thermo Fisher Scientific, United States), with absorbance ratios (A260/A280) between 1.8 and 2.0 indicating high-quality DNA. DNA integrity was verified by 1% agarose gel electrophoresis, showing distinct bands without degradation. Bisulfite transformation of genomic DNA was performed to convert unmethylated cytosines to uracils while leaving methylated cytosines unchanged, using the EZ DNA Methylation-Gold™ Kit (ZYMO Research, USA) according to the manufacturer’s protocol.

### DNA methylation analysis

**Figure 1:**
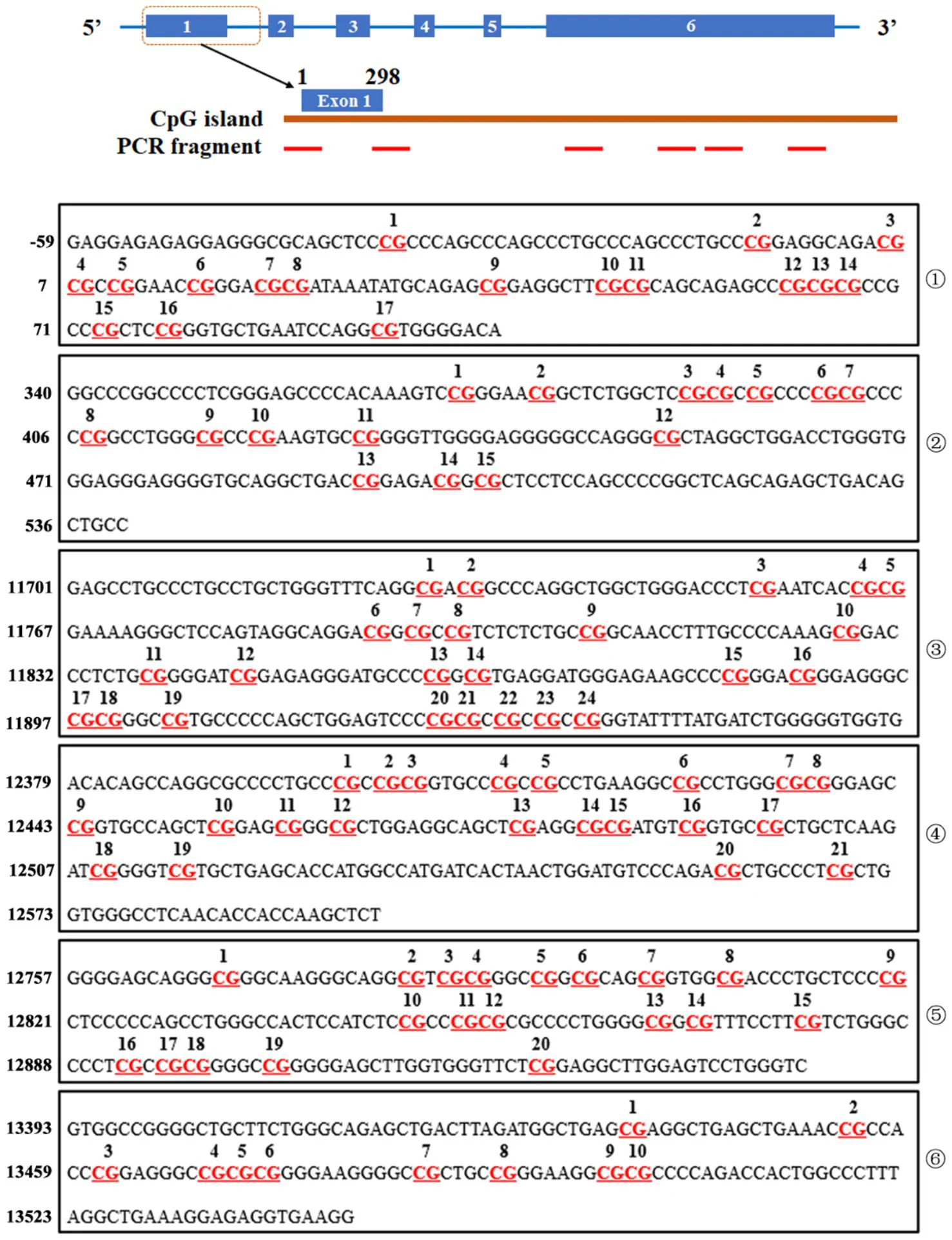
Schematic diagram of the *OLFM1* CpG island. (NCBI reference sequence: NC_000009.12).

The EBI database boss cpgplot software was used to predict CpG islands.^1^ The *OLFM1* gene (NC_000009.12) was selected as the target, with a focus on its CpG island of the first exon and its downstream region of the *OLFM1* gene (*OLFM1* CpG island) (Figure 1). The DNA methylation levels were quantified using bisulfite sequencing PCR. PCR reactions were performed in a 20 μL volume containing 10 × buffer (TAKARA, Japan), 25 mM MgCl_2_, 2.5 mM dNTPs, 1 μM of each primer, 5 U/μL HotTaq polymerase (Takara, Japan), and 1 μL bisulfite-converted DNA. PCR products were visualized on 2% agarose gels, purified using the QIAquick Gel Extraction Kit (TIANGEN, China). DNA methylation levels of six fragments of *OLFM1* were examined by next-generation sequencing-based MethylTarget^®^ (Genesky Biotechnologies Inc., China). The primer pairs used are listed in Table 2.

**Table 2:** *OLFM1* amplicon details.

Amplicon name	Primer (5′ → 3′)	Length (bp)
*OLFM1*-1	GAGGAGAGAGGAGGGYGTAGTTTT TATCCCCACRCCTAAATTCAACAC	163
*OLFM1*-2	GGTTYGGTTTTTYGGGAGTTT AACAACTATCAACTCTACTAAACCRAAACTAAAA	200
*OLFM1*-3	AGAGTTTGGTGGTGTTGAGGTT ACACAACCAAACRCCCCTACC	262
*OLFM1*-4	GGGGATATAGTTAGGYGTTTTTGTT AAACCCACCAACRAAAACAAC	219
*OLFM1*-5	GGGGAGTAGGGYGGGTAAG AACCCAAAACTCCAAACCTC	189
*OLFM1*-6	GTGGTYGGGGTTGTTTTTG CCTTCACCTCTCCTTTCAACCT	152

### Statistical analysis

All statistical analyses were performed using SPSS 22.0 (IBM Corp., United States). GraphPad Prism version 8.0 was used for plotting. Differences in demographic characteristics between the depression and healthy control groups were analyzed using independent samples *t*-tests (for normally distributed data) or Mann–Whitney *U* tests (for non-normally distributed data), and sex distribution was compared using the chi-square test. The methylation levels were analyzed using the independent sample *t*-tests. A *p*-value <0.05 was considered statistically significant. The Benjamini–Hochberg procedure was used to control the false discovery rate (FDR) for all methylation site comparisons, with a threshold set at 0.05.

## Results

### DNA methylation status of the *OLFM1* gene in patients with depression and healthy controls

In this study, the average sequencing depth of methylation sequencing samples was 1,172×, with 91.98% of the fragments having a sequencing depth >400×. As shown in Figure 1, the CpG island encompassing the first exon of the *OLFM1* gene and its downstream region was selected for analysis and divided into six regions: *OLFM1*-1 (amplicon length 163 bp, covering 17 CpG sites), *OLFM1*-2 (amplicon length 200 bp, covering 15 CpG sites), *OLFM1*-3 (amplicon length 262 bp, covering 24 CpG sites), *OLFM1*-4 (amplicon length 219 bp, covering 21 CpG sites), *OLFM1*-5 (amplicon length 189 bp, covering 20 CpG sites), and *OLFM1*-6 (amplicon length 152 bp, covering 10 CpG sites). Moreover, all detected CpG sites exhibited relatively low methylation levels, with the average methylation levels being less than 8% (Table 3).

**Table 3:** The DNA methylation levels of the *OLFM1* gene and six fragments.

Target	Mean methylation	SD
*OLFM1*-1	0.0474	0.0162
*OLFM1*-2	0.0750	0.0444
*OLFM1*-3	0.0499	0.0114
*OLFM1*-4	0.0398	0.0101
*OLFM1*-5	0.0706	0.0127
*OLFM1*-6	0.0532	0.0107
*OLFM1*	0.0558	0.0123

### Hypomethylation of the CpG islands of *OLFM1* gene in patients with depression

#### Associations of *OLFM1* gene methylation with depression

**Figure 2:**
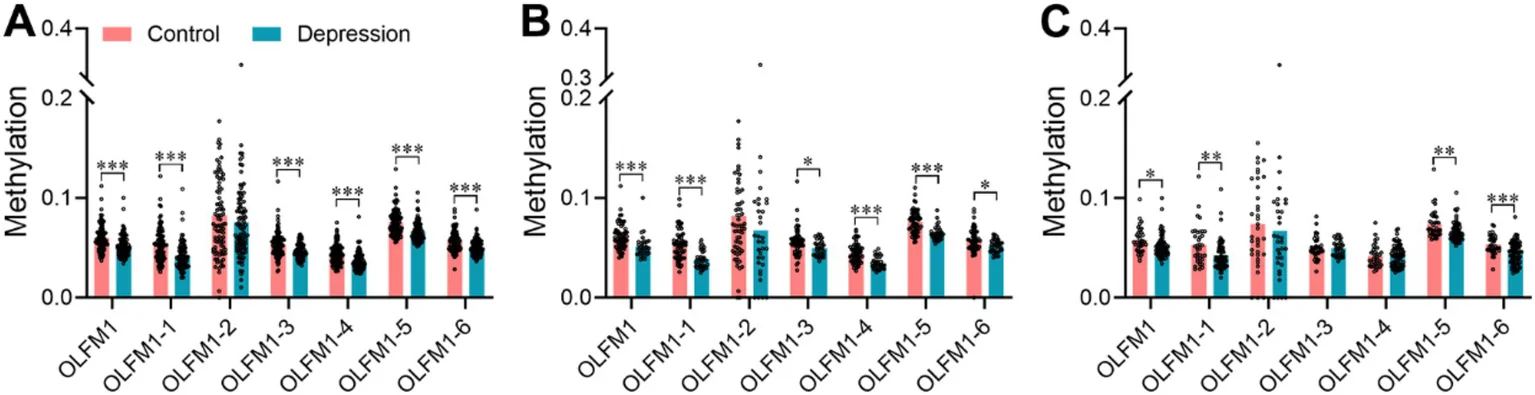
DNA methylation levels of *OLFM1* gene and 6 fragments in patients with depression (*n* = 100) and healthy controls (*n* = 100). The DNA methylation levels of total *OLFM1* gene in all samples **(A)**, male (*n*Depr_ession_ = 33, *n*Cont_rol_ = 64; **B**), and female (*n*Depr_ession_ = 67, *n*Cont_rol_ = 36; **C**) subjects. ^*^p < 0.05, ^**^p < 0.01, ^***^p < 0.001.

The *OLFM1* gene exhibited significantly lower methylation levels in the depression group compared with the control group (0.0518 ± 0.0105 vs. 0.0599 ± 0.0128; *p* < 0.001, Figure 2A). sex-stratified analysis showed that male depression patients had lower *OLFM1* methylation levels than male controls (0.0511 ± 0.0114 vs. 0.0611 ± 0.0129; *p* < 0.001, Figure 2B), which similarly to the results of female subgroup (0.0521 ± 0.0100 vs. 0.0578 ± 0.0125; *p* < 0.01, Figure 2C).

#### Methylation levels of *OLFM1* gene fragments

As shown in Figure 2A and Table 4, the average methylation levels of the five fragments (*OLFM1*-1, *OLFM1*-3, *OLFM1*-4, *OLFM1*-5, and *OLFM1*-6) were significantly lower in the depression group than in the control group (p < 0.001). Sex-stratified analysis showed that the five fragments (*OLFM1*-1, *OLFM1*-3, *OLFM1*-4, *OLFM1*-5, and *OLFM1*-6) of the *OLFM1* gene were significant hypomethylated in male depression patients compared with the male controls (*p* < 0.05, Figure 2B and Table 4). In the female subgroup, the three fragments *OLFM1*-1, *OLFM1*-5 and *OLFM1*-6 still maintained this significance (*p* < 0.01, Figure 2C and Table 4).

**Table 4:** DNA methylation levels of the *OLFM1* CpG island in patients with depression and controls.

Total	Control group (*n* = 100)		Depression group (*n* = 100)		*p*-value	FDR *q*-value
	Mean	SD	Mean	SD		
*OLFM1*	0.0599	0.0128	0.0518	0.0105	**<0.001**	**<0.001**
*OLFM1*-1	0.0530	0.0166	0.0421	0.0135	**<0.001**	**<0.001**
*OLFM1-2*	0.0801	0.0454	0.0711	0.0432	0.0976	0.0988
*OLFM1-3*	0.0535	0.0127	0.0466	0.0077	**<0.001**	**<0.001**
*OLFM1-4*	0.0435	0.0099	0.0364	0.0086	**<0.001**	**<0.001**
*OLFM1-5*	0.0756	0.0123	0.0664	0.0097	**<0.001**	**<0.001**
*OLFM1-6*	0.0566	0.0102	0.0507	0.0080	**<0.001**	**<0.001**

Male	Control group (*n* = 64)		Depression group (*n* = 33)		*p*-value	FDR *q*-value
*OLFM1*	0.0611	0.0129	0.0511	0.0114	**<0.001**	**<0.001**
*OLFM1-1*	0.0529	0.0147	0.0386	0.0088	**<0.001**	**<0.001**
*OLFM1-2*	0.0822	0.0477	0.0673	0.0595	0.0927	0.0927
*OLFM1-3*	0.0556	0.0132	0.0498	0.0078	**0.0225**	**0.0262**
*OLFM1-4*	0.0445	0.0093	0.0346	0.0061	**<0.001**	**<0.001**
*OLFM1-5*	0.0763	0.0113	0.0646	0.0071	**<0.001**	**<0.001**
*OLFM1-6*	0.0575	0.0126	0.0516	0.0065	**0.0140**	**0.0196**

Female	Control group (*n* = 36)		Depression group (*n* = 67)		*p*-value	FDR *q*-value
*OLFM1*	0.0578	0.0125	0.0521	0.0100	**0.0047**	**0.0082**
*OLFM1-1*	0.0533	0.0199	0.0439	0.0150	**0.0013**	**0.0072**
*OLFM1-2*	0.0741	0.0425	0.0730	0.0327	**0.0438**	0.0511
*OLFM1-3*	0.0500	0.0109	0.0451	0.0073	**0.0031**	**0.0072**
*OLFM1-4*	0.0419	0.0109	0.0372	0.0095	**0.0082**	**0.0115**
*OLFM1-5*	0.0742	0.0141	0.0672	0.0107	**0.0025**	**0.0072**
*OLFM1-6*	0.0535	0.0093	0.0503	0.0086	0.1311	0.1311

Bold values indicate statistically significant differences.

#### Methylation levels of individual CpG sites

**Figure 3:**
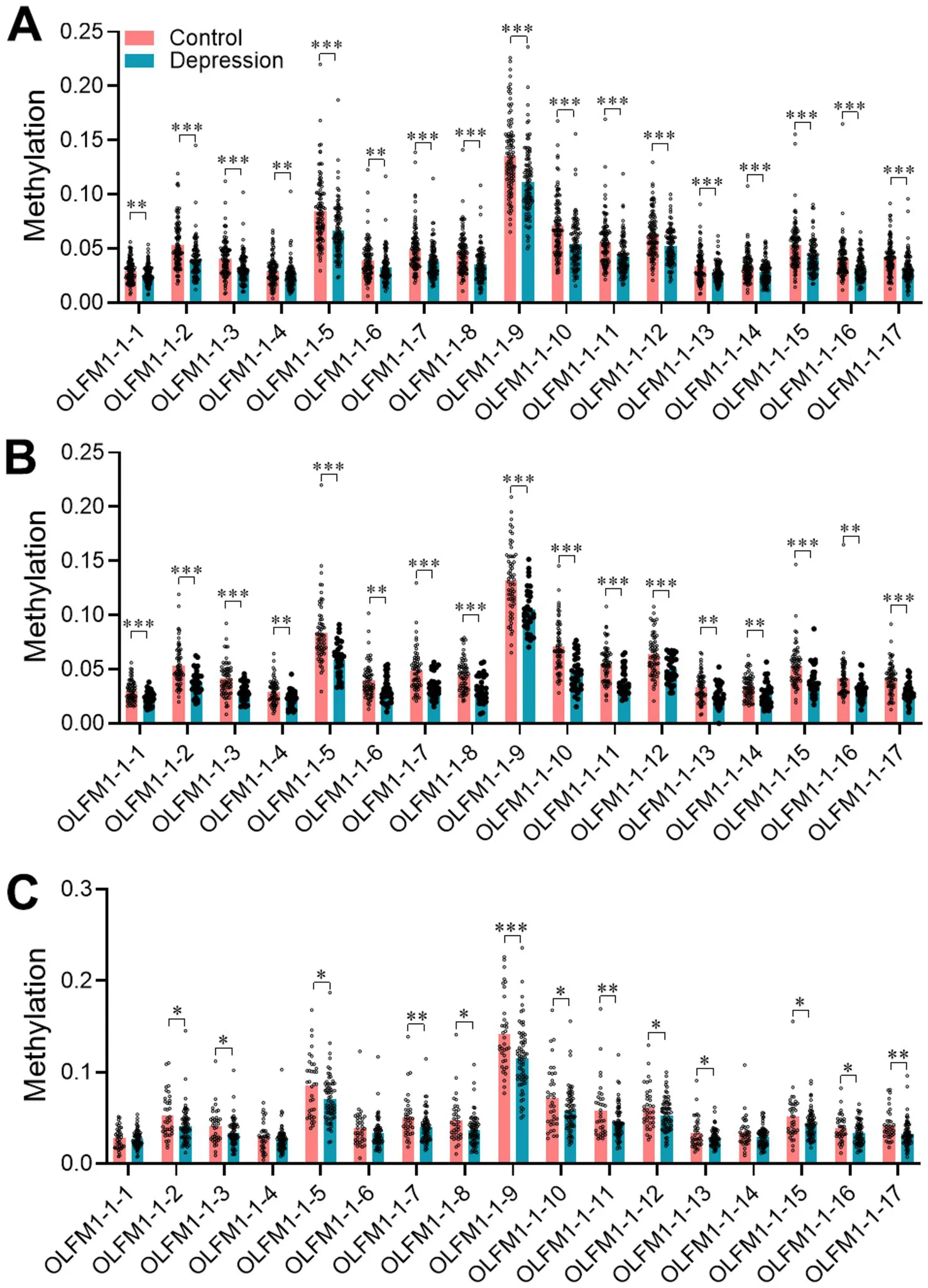
CpG site methylation levels of *OLFM1-1* in patients with depression and healthy controls. Methylation levels of 17 CpG sites in all samples **(A)**, male **(B)**, and female **(C)** subjects. ^*^p < 0.05, ^**^p < 0.01, ^***^p < 0.001.

**Figure 4:**
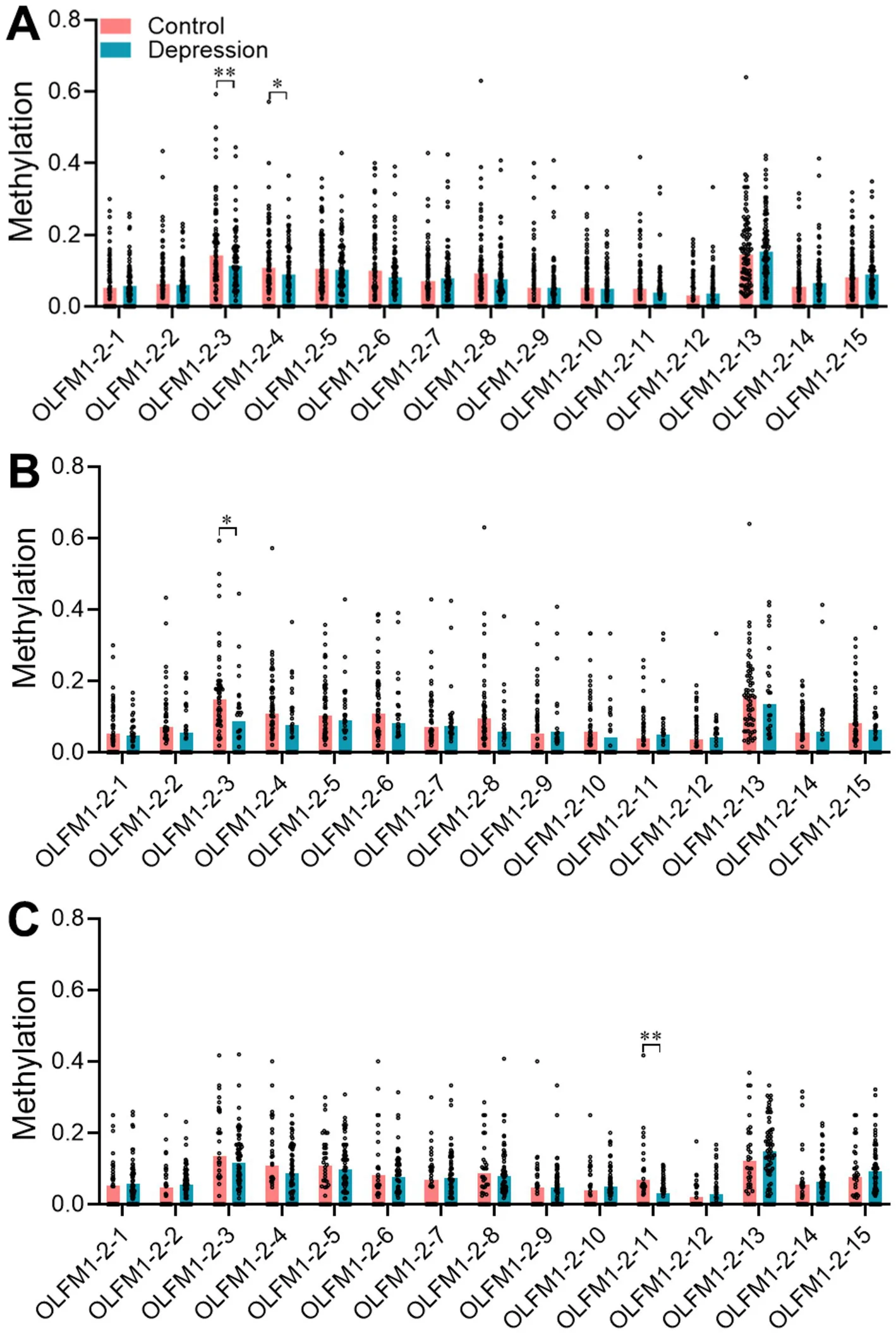
CpG site methylation levels of *OLFM1-2* in patients with depression and healthy controls. Methylation levels of 15 CpG sites in all samples **(A)**, male **(B)**, and female **(C)** subjects. ^*^p < 0.05, ^**^p < 0.01, ^***^p < 0.001.

**Figure 5:**
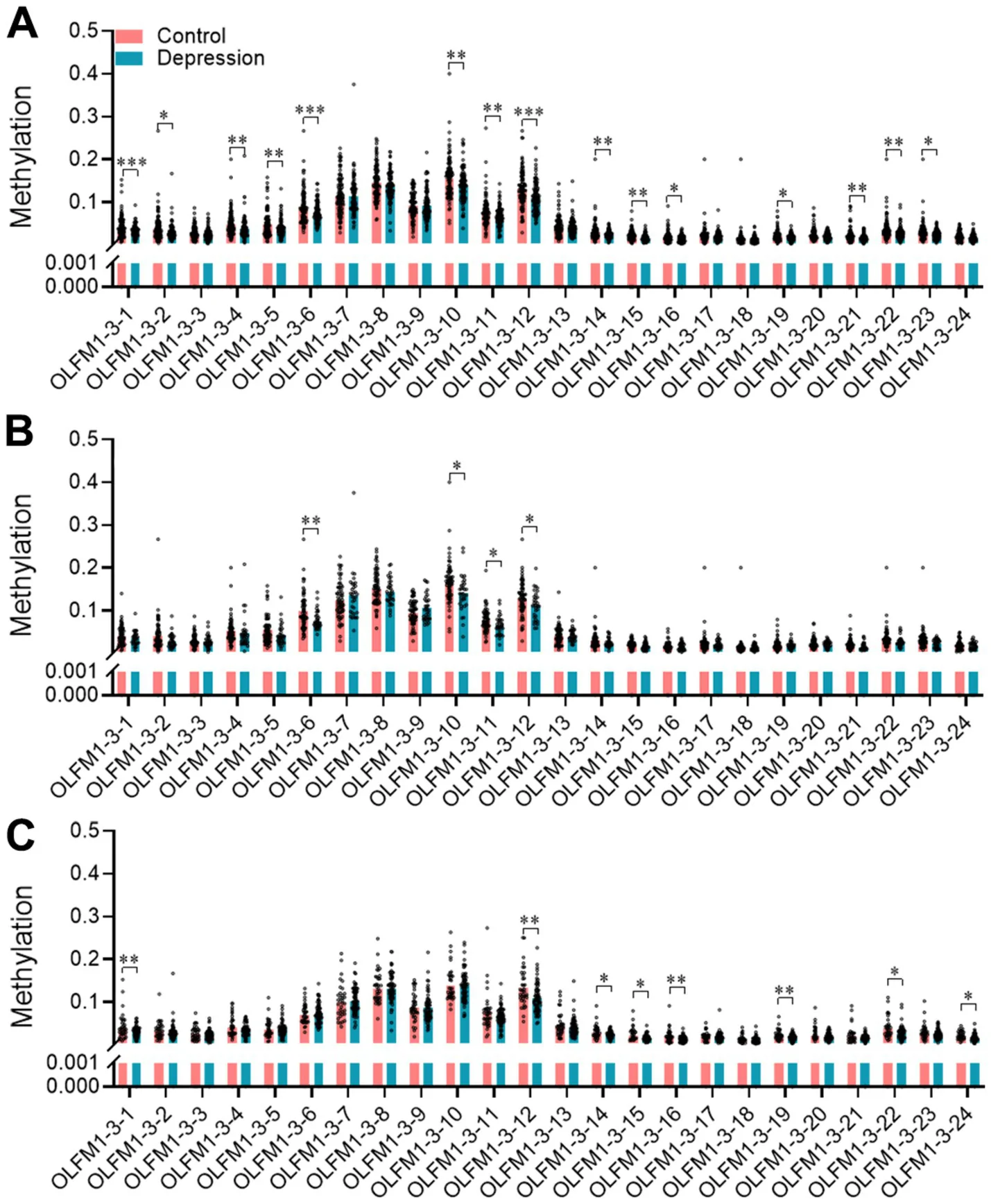
CpG site methylation levels of *OLFM1-3* in patients with depression and healthy controls. Methylation levels of 24 CpG sites in all samples **(A)**, male **(B)**, and female **(C)** subjects. ^*^p < 0.05, ^**^p < 0.01, ^***^p < 0.001.

**Figure 6:**
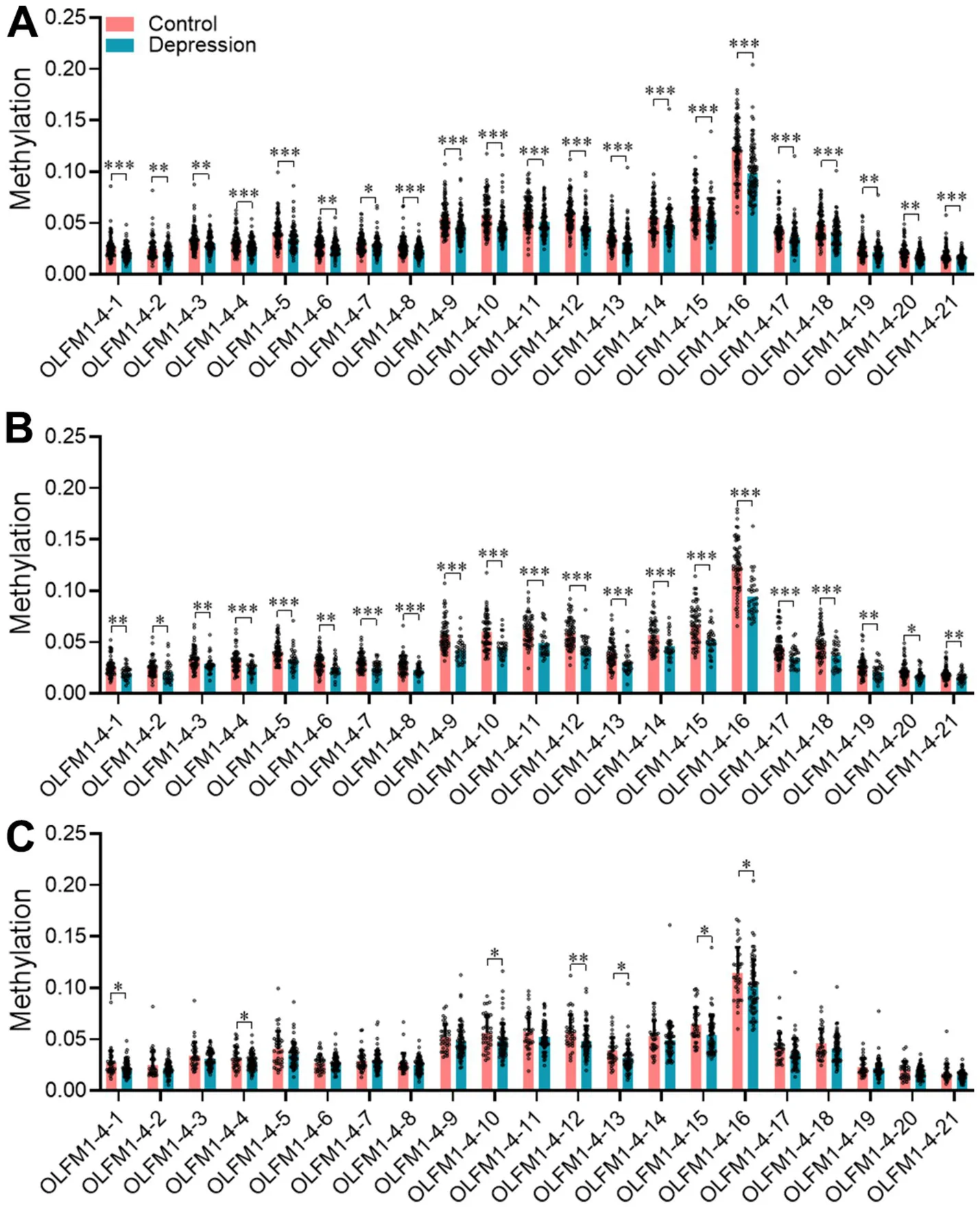
CpG site methylation levels of *OLFM1-4* in patients with depression and healthy controls. Methylation levels of 21 CpG sites in all samples **(A)**, male **(B)**, and female **(C)** subjects. ^*^p < 0.05, ^**^p < 0.01, ^***^p < 0.001.

**Figure 7:**
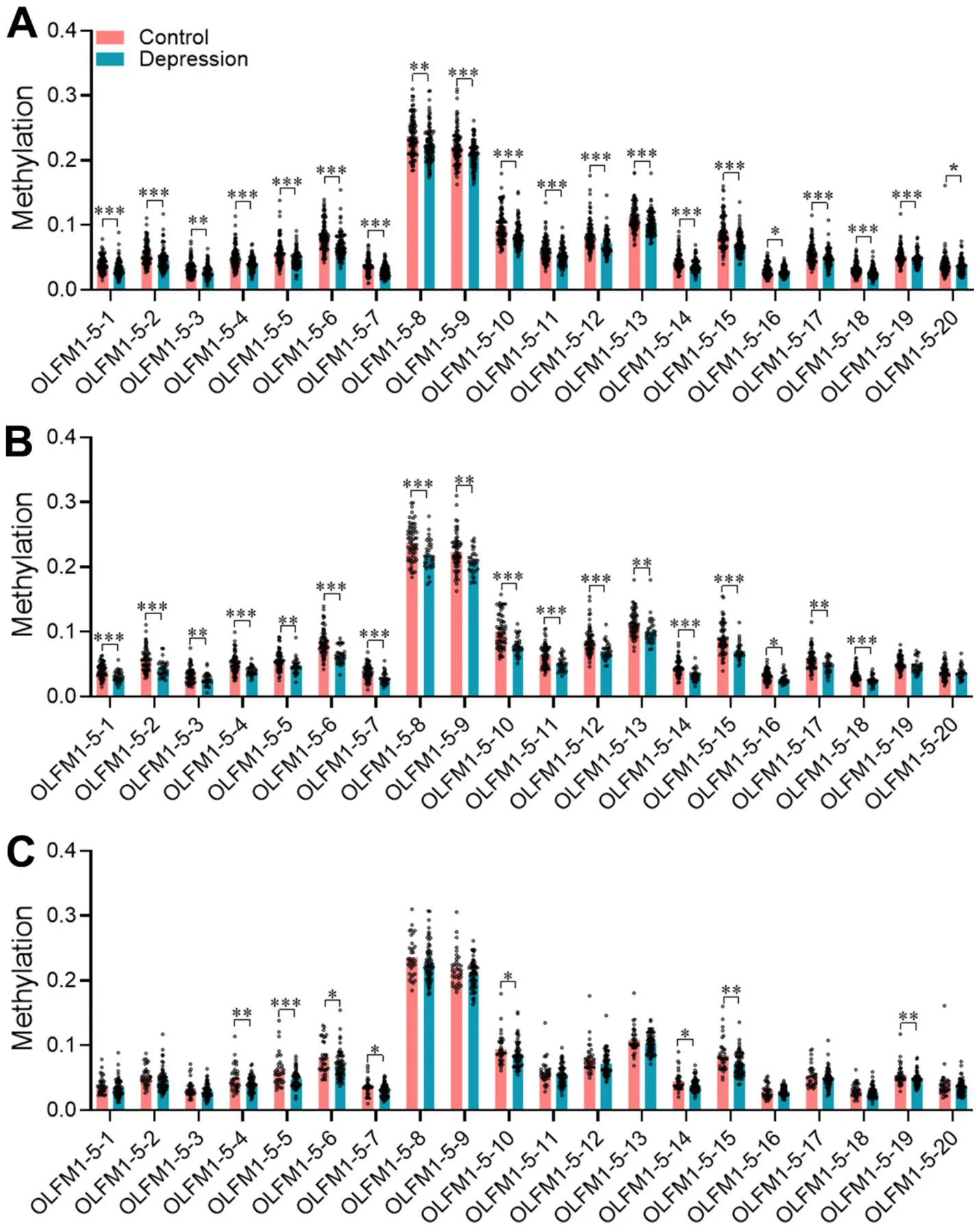
CpG site methylation levels of *OLFM1-5* in patients with depression and healthy controls. Methylation levels of 20 CpG sites in all samples **(A)**, male **(B)**, and female **(C)** subjects. ^*^p < 0.05, ^**^p < 0.01, ^***^p < 0.001.

**Figure 8:**
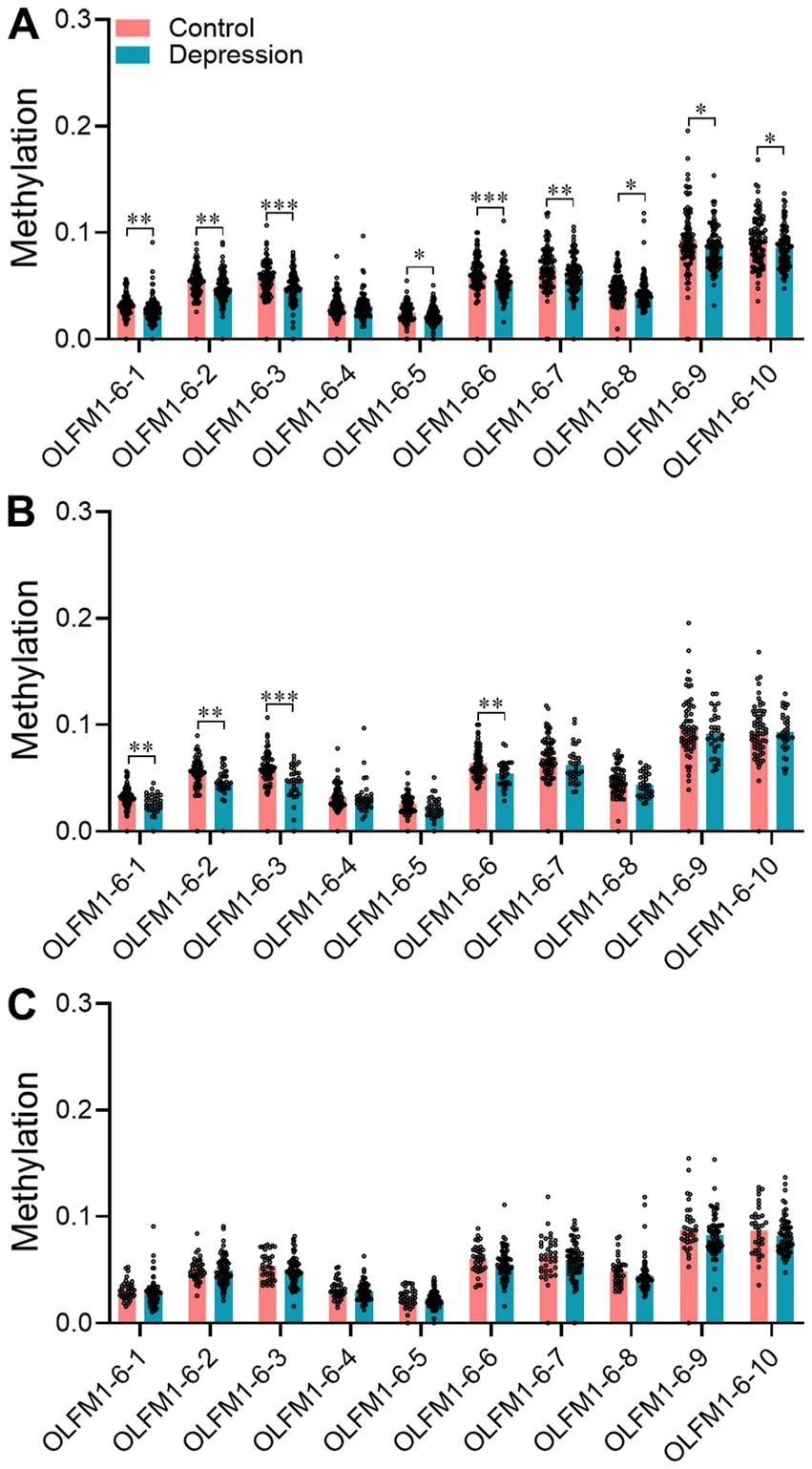
CpG site methylation levels of *OLFM1-6* in patients with depression and healthy controls. Methylation levels of 10 CpG sites in all samples **(A)**, male **(B)**, and female **(C)** subjects. ^*^p < 0.05, ^**^p < 0.01, ^***^p < 0.001.

The methylation levels of a total of 107 CpG sites were measured in the *OLFM1* CpG island. Compared with the control group, the average methylation levels of 80 CpG sites in the depression group were significantly lower than those in the control group (*p* < 0.05, Figures 3–8).

#### *OLFM1*-1

A total of 17 CpG sites were detected in the *OLFM1*-1 fragment, with the average range of methylation levels being 0.0251 ± 0.0123–0.1353 ± 0.0349 (Supplementary Table S1). Compared with the healthy control group, all CpG sites were significantly hypomethylated in the depression group (Figure 3A and Supplementary Table S1).

When grouped by sex, the male depression group had all CpG sites with significant hypomethylation compared to the healthy control group (Figure 3B and Supplementary Table S1). In the female subgroup, 13 CpG sites showed statistical significance except for *OLFM1*-1-1, *OLFM1*-1-4, *OLFM1*-1-6, and *OLFM1*-1-14 (Figure 3C and Supplementary Table S1).

#### *OLFM1*-2

A total of 15 CpG sites were detected in the *OLFM1*-2 fragment, with the average range of methylation levels being 0.0320 ± 0.0494–0.1516 ± 0.1044 (Supplementary Table S1), Among them, the methylation levels of 15 CpG sites (*OLFM1*-2-3 and *OLFM1*-2-4) in the depression group were significantly lower than those in the control group (Figure 4A).

When grouped by sex, a statistically significant increase in methylation was detected at the *OLFM1*-2-3 fragment in the depression group compared to controls (*p* < 0.05). No other fragments exhibited significant differences in methylation levels between the two groups (Figure 4B and Supplementary Table S1). In the female subgroup, a statistically significant downregulation in methylation at the *OLFM1*-2-11 fragment in the depression group relative to the control group (Figure 4C and Supplementary Table S1).

#### *OLFM1*-3

A total of 24 CpG sites were detected in the *OLFM1*-3 fragment, with the average range of methylation levels being 0.0111 ± 0.0079–0.1560 ± 0.0504 (Supplementary Table S1). Among them, the methylation levels of 15 CpG sites (*OLFM1*-3-1, *OLFM1*-3-2, *OLFM1*-3-4, *OLFM1*-3-5, *OLFM1*-3-6, *OLFM1*-3-10, *OLFM1*-3-11, *OLFM1*-3-12, *OLFM1*-3-14, *OLFM1*-3-15, OLFM1-3-16, *OLFM1*-3-19, *OLFM1*-3-21, *OLFM1*-3-22, and *OLFM1*-3-23) in the depression group were significantly lower than those in the control group (Figure 5A and Supplementary Table S1).

When the samples were grouped by sex, in the male group, there were 4 CpG sites (*OLFM1*-3-6, *OLFM1*-3-10, *OLFM1*-3-11, and *OLFM1*-3-12) that showed significant hypomethylation in depression (Figure 5B and Supplementary Table S1). In the female group, 8 CpG sites (*OLFM1*-3-1, *OLFM1*-3-12, *OLFM1*-3-14, *OLFM1*-3-15, *OLFM1*-3-16, *OLFM1*-3-19, *OLFM1*-3-22, and *OLFM1*-3-24) showed significant hypomethylation in depression (Figure 5C and Supplementary Table S1).

#### *OLFM1*-4

A total of 21 CpG sites were detected in the *OLFM1*-4 fragment, with the average range of methylation levels being 0.0152 ± 0.0050–0.1201 ± 0.0257 (Supplementary Table S1). All CpG sites in the depression group were significantly hypomethylated compared with the healthy control group (Figure 6A and Supplementary Table S1).

After sex stratification, all CpG sites in the male subgroup showed lower methylation levels in the depression group compared with the healthy control group (Figure 6B and Supplementary Table S1). Among females, the methylation levels of 7 CpG sites (*OLFM1*-4-1, *OLFM1*-4-4, *OLFM1*-4-10, *OLFM1*-4-12, *OLFM1*-4-13, *OLFM1*-4-15, and *OLFM1*-4-16) were statistically significant (Figure 6C and Supplementary Table S1).

#### *OLFM1*-5

A total of 20 CpG sites were detected in the *OLFM1*-5 fragment, with the average range of methylation levels being 0.0270 ± 0.0084–0.2374 ± 0.0282 (Supplementary Table S1). All CpG sites in the depression group were significantly lower than those in the healthy control group (Figure 7A and Supplementary Table S1).

In the male subgroup, 18 CpG sites, with the exceptions of *OLFM1*-5-19 and *OLFM1*-5-20, were significantly hypomethylated in the depression group compared with the healthy control group (Figure 7B and Supplementary Table S1). In the female subgroup, the methylation levels of 8 CpG sites (*OLFM1*-5-4, *OLFM1*-5-5, *OLFM1*-5-6, *OLFM1*-5-7, *OLFM1*-5-10, *OLFM1*-5-14, *OLFM1*-5-15, and *OLFM1*-5-19) were significantly lower in the depression group than those in the healthy control group (Figure 7C and Supplementary Table S1).

#### *OLFM1*-6

A total of 10 CpG sites were detected in the *OLFM1*-6 fragment, with the average range of methylation levels being 0.0222 ± 0.0086–0.0943 ± 0.0278 (Supplementary Table S1). Among them, 9 CpG sites (*OLFM1*-6-1, *OLFM1*-6-2, *OLFM1*-6-3, *OLFM1*-6-5, *OLFM1*-6-6, *OLFM1*-6-7, *OLFM1*-6-8, *OLFM1*-6-9, and *OLFM1*-6-10) were significantly hypomethylated in the depression group compared with the control group (Figure 8A and Supplementary Table S1).

After stratifying all the samples by sex, 4 CpG sites (*OLFM1*-6-1, *OLFM1*-6-2, *OLFM1*-6-3, and *OLFM1*-6-6) were significantly hypomethylated in the male depression group (Figure 8B and Supplementary Table S1). In the female subgroup, the average range of methylation levels at CpG sites showed no significant differences in both groups (Figure 8C and Supplementary Table S1).

## Discussion

A study reported that *OLFM1* may serve as a candidate gene for neuropsychiatric disorders (15). Moreover, epigenome-wide association studies have indicated that abnormal gene expression was closely associated with the occurrence and development of various mental disorders, including depression (26). For example, abnormal DNA methylation was observed in depression animal models and depressed individuals (27). Notably, DNA methylation sequencing has been extensively validated as a feasible and robust tool for investigating the epigenetic mechanisms underlying depression, supported by three core advantages: first, DNA methylation is a stable, heritable epigenetic modification that persists in somatic cells, enabling the capture of long-term pathophysiological changes in depression rather than transient transcriptional fluctuations (28); second, advances in sequencing technologies [e.g., targeted high-throughput methylation sequencing, whole-genome bisulfite sequencing (WGBS)] have achieved high-resolution and high-throughput detection of CpG site methylation, with excellent reproducibility in both small and large cohorts (29); third, numerous EWAS have successfully identified depression-associated methylomic signatures using these technologies, such as differential methylation in stress response pathways (e.g., *NR3C1*, *FKBP5*) and neuroplasticity-related genes, confirming the technical feasibility of DNA methylation sequencing in decoding depression’s epigenetic landscape (30, 31).

This is the first study to examine the DNA methylation levels of the *OLFM1* gene in human peripheral blood from depression patients and healthy controls, which will fill the knowledge gap in research on *OLFM1* gene methylation in patients with depression. The use of peripheral blood for DNA methylation analysis in depression research is further justified by compelling evidence of feasibility and representativeness: peripherally circulating leukocytes are easily accessible via non-invasive sampling, facilitating large-scale clinical sample collection and longitudinal follow-up, which is critical for epidemiological and translational studies (32). More importantly, accumulating studies have verified that peripheral blood methylation profiles can partially recapitulate epigenetic alterations in the brain. For instance, genome-wide methylation correlation analysis between peripheral blood and postmortem brain tissues (e.g., hippocampus, prefrontal cortex) has revealed conserved methylation patterns in key depression-related genes (e.g., *NR3C1*), with overlapping differential methylation regions that are functionally relevant to neuronal function and stress response (33). In the present study, our data showed that the DNA methylation levels of the *OLFM1* gene were lower in patients with depression than in healthy controls, especially in five fragments (*OLFM1*-1, *OLFM1*-3, *OLFM1*-4, *OLFM1*-5, and *OLFM1*-6) of the *OLFM1* CpG island. Among the total 107 CpG sites in six fragments of the *OLFM1* gene, 84 CpG sites showed low methylation in patients with depression. Lyn et al. (34) reported that nominally differentially methylated CpG in *OLFM1* gene involved in synaptic signaling and neurodevelopment.

Numerous studies have demonstrated that DNA methylation has been implicated in the pathophysiology of depression (35, 36). For instance, peripheral blood DNA methylation levels of *BDNF*, *COMT*, and *SLC6A4* genes were closely associated with acute clinical symptoms of anxiety and depression (37). Representative studies further confirm the validity of peripheral blood methylation sequencing: a large-scale EWAS involving 1,234 major depressive disorder (MDD) patients and 1,575 controls identified hypomethylation of the *FKBP5* gene in peripheral blood, which was replicated in an independent cohort and correlated with MDD susceptibility and treatment response (38). Webb et al. (39) conducted a systematic review and meta-analysis confirming that peripheral blood methylation of *BDNF*, *SLC6A4*, *HTR1A*, *HTR1B*, and *IL11* genes are consistently associated with antidepressant response, providing robust evidence for the representativeness of peripheral blood epigenetic markers. Starnawska et al. (40) found that the DNA methylation levels of KLK8 in the blood correlated with the severity of depressive symptoms, yet not with the diagnosis of depression. Of note, increased DNA methylation served as a risk factor for suicide in depression (41). However, the functional role and mechanism of *OLFM1* methylation in the progression of depression have not been reported. Barrera et al. (42) reported that alterations in the DNA methylation of *OLFM1* were associated with bovine blastocysts. Functionally, emerging studies have shown that *OLFM1* was a secreted glycoprotein predominantly expressed in the central nervous system that plays critical roles in neurodevelopment, synaptic plasticity, and neuronal survival (10). Emerging research suggests a potential link between dysregulated *OLFM1* expression and a spectrum of neuropsychiatric disorders. These disorders include multiple sclerosis, amyotrophic lateral sclerosis, mania, depressive disorder, Tourette syndrome, obsessive-compulsive disorder, and attention-deficit/hyperactivity disorder (15, 43, 44). Another study reported that knockdown of *OLFM1* promoted brain dystrophy and abnormal behavior by promoting neonatal death and synaptic dysfunction (45). Other studies have confirmed that *OLFM1* overexpression inhibited the growth and metastasis of cancer cells (46–48). Additionally, given *OLFM1*’s interaction with the Nogo A receptor complex and its role in axon growth, dysregulated *OLFM1* expression could contribute to the synaptic and structural brain changes observed in depression (49).

Moreover, previous studies have confirmed that demographic characteristics (50) (e.g., education, marital status, alcohol consumption, tobacco smoking, body mass index) and sex differences (51) could affect the prevalence of psychiatric disorders. As expected, our findings revealed that education and marital status exerted a significant influence on the *OLFM1* methylation level in both depression patients and healthy controls (Supplementary Table S2). Interestingly, our results also revealed that six fragments of the *OLFM1* gene containing 65 CpG sites were hypomethylated in depressed males compared to controls, but three of those fragments (*OLFM1*-1, *OLFM1*-5, and *OLFM1*-6) and 37 CpG sites across the six regions exhibited hypomethylation in depressed females. Existing studies have found that the incidence of depression was higher in females than in males (52, 53), but the suicide rate in males was 3 or 4 times higher than in females (54). On the contrary, other studies reported that females respond better to antidepressants than males (55). These data indicated that sex served as a critical factor in depression pathogenesis, and sex-differentiated methylation of *OLFM1* may provide insights into its regulatory role in depression. Similarly, a recent study reported that the DNA methylation levels of *OLFM1* were higher in patients with treatment response to selective serotonin reuptake inhibitors than those in non-response among patients with major depressive disorder (34). Importantly, the cross-sectional nature of our study precludes determination of the temporal relationship between *OLFM1* hypomethylation and depression. It remains unclear whether reduced methylation at *OLFM1* represents a predisposing factor that increases vulnerability to depression, a consequence of the neurobiological changes associated with depressive illness, or an epiphenomenon related to unmeasured confounders. Longitudinal cohort studies tracking methylation dynamics before, during, and after depressive episodes, combined with functional validation in experimental models, will be essential to clarify the causal role, if any, of *OLFM1* methylation in depression pathogenesis.

In summary, this study confirmed that the methylation levels of the *OLFM1* gene promoter in the peripheral blood were lower in patients with depression (particularly males) than those in healthy controls, which indicated that the hypomethylation of *OLFM1* may contribute to the pathogenesis of depression. The feasibility of DNA methylation sequencing in depression research, combined with the well-established representativeness of peripheral blood as a surrogate tissue, strongly supports the validity of our findings. Moreover, this study addresses a gap in research on epigenetic modifications of the *OLFM1* gene in depression. Follow-up investigations will validate the pathogenic association between *OLFM1* CpG methylation and major depressive disorder using human brain tissue specimens, and delineate the molecular regulatory cascades governed by *OLFM1* in depression through *in vitro* cellular assays and *in vivo* animal models. Meanwhile, the mRNA and protein level of *OLFM1* should be detected and quantified. These findings will contribute to a deeper understanding of the pathophysiological of depression and may provide new biomarkers for the treatment and diagnosis of depression.

## Data Availability

The original contributions presented in the study are included in the article/Supplementary material, further inquiries can be directed to the corresponding authors.

## Footnotes

1
http://emboss.bioinformatics.nl/cgi-bin/emboss/cpgplot
